# Micro-/Nano-Fiber Sensors and Optical Integration Devices

**DOI:** 10.3390/s22197673

**Published:** 2022-10-10

**Authors:** Jin Li

**Affiliations:** 1 College of Information Science and Engineering, Northeastern University, Shenyang 110819, China; lijin@ise.neu.edu.cn; 2 Hebei Key Laboratory of Micro-Nano Precision Optical Sensing and Measurement Technology, Qinhuangdao 066004, China

## 1. High-Performance Micro/Nanofiber Sensors

Because of their strong surface evanescent field, micro-/nanofibers have been used to develop optical sensors and modulation devices with a high performance and integration. In recent years, they have become an important branch of optical fiber optics and novel sensors, and have received extensive attention from researchers from all over the world. On the one hand, the sensing and optical properties of micro/-nanofiber devices can be optimized by introducing different micro/nanostructures through micro/nano-processing technology (femtosecond laser processing, electron beam etching, ion beam engraving, and chemical etching) or through the functionalization of new nanomaterials (film coating and particle doping) in order to achieve high selectivity detection for the desired targets. On the other hand, their tight confinement effect for light based on the high evanescent field coupling efficiency is also conducive to the integration of free-standing optical fibers with typical planar micro-/nanostructures on silicon substrates, making them a promising candidate for exploring nanophotonic integrated devices.

The development of micro/nanofiber sensors and the related integrated systems is a grand project spanning photonics, engineering, and materials science, and will become a hot academic research field. During the development of miniature optical sensors, different materials and micro/nanostructures are reasonably designed and functionalized on ordinary single-mode optical fibers. The combination of various special optical fibers and new micro/nanomaterials has greatly improved the performance of the sensors. In terms of optical integration, micro/nanofiber plays as an independent and movable optical waveguide device and can be conveniently integrated into the two-dimensional chip to realize the efficient transmission and information exchange of optical signals based on optical evanescent field coupling technology. In terms of systematic integration, the unique optical transmission mode of optical fiber shows its great potential in the array and networking of multiple sensor units.

In this book, more than ten research papers were collected and studied on the optical micro/nanofiber devices and related integrated systems, covering the high-performance optical micro/nanofiber sensors, fine characterization technologies for optical micro/nanostructures, weak signal detection technologies in photonic structures, as well as the fiber-assisted highly integrated optical detection systems.

Using the low-cost optical fiber fusion splicers and common single-mode optical fibers, many optical fiber composite structures can be fabricated, such as the fusion joints with offset core, spherical convex cones with enlarging waist, microfiber tapers with the biconical region, microfiber couplers with ultra-thin connecting zone and novel fibers with special materials or structures, which have been designed to develop the compact fiber sensors with high performance.

Deng et al. designed a Mach-Zehnder interferometer using the dislocation fusion technology [1] and modified its surface with 3-aminopropyl-triethoxysilane (APES), which plays as a typical polymer to provide the electrostatic bonding chemical bonds during the layer-by-layer assembly technology for elaborating the optical fibers. The morphological parameters of the APES coating layer were determined and optimized by the microstructural Mach-Zehnder fiber interferometer This technology is crucial for the functional modification of selective enzymes, antibodies, or chemical bonds on the surface of optical fibers. This process is indispensable in the development of fiber biochemical sensors. In this work, the effects of different concentrations, dripping time, and dosage of APES on the transmission spectrum were studied. The best extinction ratio has been experimentally demonstrated to be ~1.165 for the corresponding processing parameters of 2% (concentration), 1 h (immersing time), and 1.5 mL (dosage).

Yi et al. Proposed a convex micro-cone structure for vectorization monitoring of curvature [2]. In this optical fiber structure, the size of the convex cone is very small, which is not different from the ordinary single-mode optical fiber in appearance. A pair of convex micro-cones were used to construct a multimode interferometer. The effects of different overlap lengths and interference region lengths on the interference spectrum were studied to optimize the structural parameters. The curvature responses and dynamic changes in two orthogonal directions have been real-time determined with the sensitivities of 79.1°/m^−1^ and −48.0°/m^−1^, corresponding to the resolutions of 2.69 × 10^−3^ m^−1^ and 4.44 × 10^−3^ m^−1^, respectively. The simple and flexible structure, as well as the array and networking advantages of the optical fiber, make this structure promising for real-time monitoring of the direction and extent of curvature changing at multiple points.

Xiang et al. proposed stretchable optical fiber sensors (SPFSs) by encapsulating the dual-cone microfiber in the polydimethylsiloxane (PDMS) membrane [3]. This sensor can be attached to the human skin as a wearable healthcare device to measure the strain, temperature, and humidity caused by pulse, body temperature, and respiration. In this work, the bending and recovering processes have been experimentally monitored to reveal the wrist pulse of ~68 beats per minute. The temperature sensitivity of 0.02 dBm/°C was verified during 30~40 °C when the sensor was fixed on the back of the hand. The proposed sensor was also packaged in a mask to determine the high humidity with a sensitivity of 0.5 dB/%RH.

Gao et al. attached an optical nanofiber coupler to a diaphragm to determine the acoustic in the range of 30~20,000 Hz [4]. The highest sensitivity of 1929 mV/Pa was experimentally demonstrated at the frequency of 120 Hz with a high signal-to-noise ratio of 42.45 dB. The lowest detection limit was verified to be 330 µPa/Hz^1/2^. This acoustic vibration sensor has great application potential in low-frequency vibration ranges, such as seismic wave detection.

The new special optical fiber can be developed to meet some requirements of the optical communication and sensing applications in some extreme environments. Zhang et al. doped different metal oxide materials (Al_2_O_3_, Y_2_O_3_, and P_2_O_5_) into the fiber core to build a specific fiber [5]. They etched the fiber grating structure on the independently designed fiber, and experimentally verified its sensing characteristics to curvature and temperature. The bending sensing sensitivity is 21.85 dB/m^−1^ in the range of 0~1.2 m^−1^, and the corresponding long-term working fluctuation error is only 0.014 m^−1^. In the high-temperature range of 20~1000 °C, the temperature response sensitivity is 14.1 pm/°C, and the response time is 0.6 s, when the temperature jumped to 955 °C.

## 2. Fine Characterization of Micro/Nanostructures

The research and analysis of the morphological parameters and material composition of different micro/nanostructures are also crucial for developing highly integrated sensor devices. The resonance spectrum of optical signals can be generated in the periodic microstructures, which are expected to be applied to the precise identification of biological tissues.

Yang et al. combined ultrasonic (US) and photoacoustic (PA) spectroscopy technology to realize the detection of the periodic microstructure of bone samples [6], where the standing waves were generated among the pores. The US resonance spectra of bone samples after demineralization and decollagenization have been compared and analyzed. It provides a promising technique to early determine osteoporosis.

Brillouin optical time domain analysis (BOTDA) has been widely concerned to monitor the structural health of bridges, dams, tall buildings, equipment, and pipelines. It is worth mentioning that some minor defects can also be detected, as Yuan et al. verified in their work [7], where the micro-cracks with a width of 2~9 μm were accurately measured by combining theoretical models and experiments based on BOTDA technology. Brillouin peak will be significantly increased with the enlarging size of defects. This work proposed an effective method to simulate the strain distribution along the optical fiber due to the micro-cracks.

## 3. Weak Optical Signal Detection in Photonic Structures

The detection and accurate acquisition of weak optical signals are extremely important for micro/nanoscale photonic devices. Liu et al. designed the cryogenic system with a fluctuation of ±0.3 °C to smoothly cool down the working temperature of a silicon photomultiplier (SiPM) and compared its dynamic performance in operating voltage and dark noise with those at room temperature [8]. The dark count rate was reduced for 6 orders from 120 kHz/mm^2^ to 0.1 Hz/mm^2^.

Yang et al. proposed an off-axis Fresnel zone plate based on double exposure points holographic surface interference to eliminate the aberration in recording the instantaneous three-dimensional imaging [9]. The corresponding impact of the introduced method on the Seidel coefficient, average gradient, and signal-to-ratio has been compared. The imaging performance has been significantly improved and verified by many parameters, such as spherical aberration W040, coma aberration W131, image dispersion W222, field curvature W220, average gradient, and signal-to-noise ratio.

## 4. High Integration Optical Fiber Assisted-Sensing System

The optical fiber can be conveniently connected to the high-performance optical system to achieve the efficient transmission and collection of optical signals, so as to improve the integration density of laser detection devices.

Hu et al. designed an optical fiber integrated dissolved gas detector to real-time monitor the dissolved carbon dioxide in seawater based on ring cavity down spectroscopy [10]. The sensor effectively improves the water gas separation efficiency, and the detection rate is 10 times higher than that of the ordinary sensing method. The practical test of offshore seawater shows the practicability of the proposed sensor system, where real-time monitoring was achieved for the water-soluble carbon dioxide with a concentration of ~950 ppm. The sensor system is highly adaptable to the complex environment of the deep sea (the depth of <4500 m and low temperature) for a long time.

Zhou et al. used the Sagnac microfiber loop to measure the concentration of airborne molecular contaminants (AMCs) in the air environment, which becomes a serious threat to the service life of UV high-energy lasers [11]. The mesoporous silica was elaborated on the surface of the Sagnac ring by microheater brushing method and dip coating technique. It can absorb the AMCs to cause a change in its effective refractive index. The sensitivity was determined to be 0.11 nm (mg/m^3^). This work supplies a promising way to detect trace contaminates in the air environment around the lens or micro/nanostructures.
